# Determination of the diffusion coefficient through oil absorption and moisture loss, such as the porosity of pieces of yam (*Dioscorea rotundata*) during deep fat frying

**DOI:** 10.1016/j.heliyon.2021.e08036

**Published:** 2021-09-22

**Authors:** Arrazola Guillermo, Alvis Armando, Romero Pedro

**Affiliations:** Food Engineering Program, Faculty of Engineering, University of Cordoba, Research Group in Vegetable Processes and Agroindustry, Colombia

**Keywords:** *Dioscorea rotundata*, Fick's law, Diffusion coefficient, Porosity, Deep fat frying

## Abstract

This study evaluated moisture loss, oil gain and porosity when frying pieces of yam (*Dioscorea rotundata*). The parallelepiped-shaped samples, approximately 1 × 1 × 4 cm, were subjected to frying temperatures of 145, 165 and 185 °C for 50, 150, 300, 450, or 600 s. Fick's law was used to determine the diffusion coefficient from the experiment data for the varieties 153 traditional Espino and 125 Brazilian Espino. The moisture loss in 153 traditional Espino was greater than in 125 Brazilian Espino. The diffusion coefficient and the activation energy were determined for both varieties, which were higher in the 153 traditional Espino variety. The porosity was expressed as a percentage and was also higher in the 153 traditional Espino variety.

## 1 Introduction

Yam is a tropical plant of African and Asian origin. It belongs to the *Dioscoreales* order and the *Dioscoreaceas* family, which contains 6 genera, of which *Dioscorea* is the most important with 600 identified species, but only 12 species are edible (Vidal, 2012). There are numerous yam varieties (*Genus Dioscorea*), some of which are indigenous to Africa, Asia and the Americas. They vary in terms of color, size, cooking quality, leaf structure, and palatability (Latham, 2002). In Colombia, yam production is concentrated in the departments of the Caribbean region, where its consumption is also concentrated (Reina, 2012). The Caribbean region contributes more than 90% of domestic production, distributed in the Departments of Córdoba (8,117 tons), Sucre (18,594 tons), Bolívar (113,620 tons) and César (7,382 tons). The hawthorn, diamond and criollo varieties are the more important ones because of their cultivated area and greater local demand (FAO, 2012). Other departments, such as Antioquia, Chocó, Casanare and Vaupés have minimal participation (Reina, 2012).

Today, frying is one of the more popular food preparation procedures; it is quick and develops desirable flavor and texture. Frying products rich in starches (as in this case) has been studied mainly for potatoes, cassava and yams (Alvis et al., 2008). A clear understanding of yam frying processes is essential for the research and development of new products (snacks) and quality control of raw materials and intermediate and finished products (Pacheco et al., 2020).

Frying is a simultaneous mass and heat transfer operation, where cooking in oil-fat imparts various desirable quality attributes, such as taste, texture, appearance and odor, to food products. These desirable changes in foods include a combined effect of crusting, moisture loss, protein denaturation, oil-fat gain, starch gelatinization, and large, internal microstructural changes (Saguy and Pinthus, 1995; Saguy et al., 1998). Two principals are seen in dehydration processes: one is water transfer without a change of state (osmotic dehydration), and the other includes a change of state (vaporization or sublimation).

In this research, two varieties (153 Espino and 125 Brazilian Espino) with a low content of reducing sugars were chosen to study the effect that frying produces on moisture loss, oil gain, and porosity in pieces of yam with different temperatures and frying times, along with determining the effective diffusion coefficient and activation energy.

## 2 Materials and methods

### 2.1 Selection of plant materials

Rhizomes of Creole yam (*Dioscorea rotundata*) were selected, which were acquired from the Germplasm Bank of the University of Cordoba (Colombia) and which came from the Atlantic Coast region. A sample of 10 kg was collected from each variety, with similar sizes and weights. The yams were harvested manually and according to their plot number for the varieties 125 Brazilian Espino, 153 Traditional Espino, 091 Espino and 619 African Espino. The yams were preserved whole at room temperature and humidity during this research. All units of each sample were washed, peeled and cut with a “potato-cutter®” into parallelepiped shapes, approximately 1 × 1 × 4 cm.

#### 2.1.1 Chemical properties

The samples were dried in an oven with temperature control following AOAC method number 925.09. The content of fat, protein, ash and fiber were determined in triplicate in the dry samples following the methods of AOAC numbers 920.39, 920.87, 923.03 and 962.09, respectively (AOAC, 2003). The remaining samples were stored for 90 days at 28 °C. The total carbohydrates were estimated with differences. Additionally, triplicate analyses of reducing sugars were performed according to the calorimetric method with Dinitrosalicylic Acid. The varieties with a low content of reducing sugars when left in the environment were selected for the frying process, which were the ones that took longer to suffer enzymatic browning (Alvis et al., 2008).

### 2.2 Determination of the diffusion coefficient, activation energy and mass porosity

Pieces of yam (1 × 1 × 4 cm) were immersed in a mixture of vegetable oil with different frying temperatures (145, 165 and 185 °C) (soybean and palm olein), the ARO brand manufactured by Duquesa SA, using a two-compartment GSM® brand stainless steel fryer with a temperature control system. After different times (50, 150, 300, 450, 600 s), the samples were removed and placed on filter paper to remove the superficial oil to immediately determine the moisture at 105 °C until constant weight (AOAC, 2003). The oil content was determined with solvent extraction using hexane for 8h (AOAC, 2003). For each frying temperature, 3 replications were carried out.

The moisture content data for the pieces of yam as a function of time were used to determine the diffusion coefficient by applying Fick's second law for unidirectional diffusion along the (x) axis with Eq. (1):

(1) $$\frac{\partial \text{C}}{\partial \text{t}}=D_{\text{a}}\frac{\partial^{2}\text{C}}{\partial^{2}{\text{x}}^{2}}$$

Vélez-Ruiz and Sosa-Morales (2003) and Gamble et al. (1987) used Fick's diffusion law to model moisture loss with a zero-order kinetic model to predict oil absorption. Math et al. (2004) studied the mechanisms of moisture transfer and oil absorption during potato frying using a first-order kinetic model. Previous theories agree that fat absorption and water loss occur during deep frying. In the tests, the piece of yam had a parallelepiped shape, 1 × 1 × 4cm. Therefore, the geometry was assumed as that of a plate, and, since the diffusion was unstable, Eq. (2) was used:

(2) $$\frac{{\text{C}}_{t}-{\text{C}}_{\infty }}{{\text{C}}_{i}-{\text{C}}_{\infty }}=\frac{8}{{\text{π}}^{2}}\left(e^{-\left(\frac{{\text{π}}^{2}}{4}\right)\left(\frac{{\text{D}}_{\text{a}}\text{t}}{{\text{L}}^{2}}\right)}+\frac{1}{9}{\text{e}}^{-9\left(\frac{{\text{π}}^{2}}{4}\right)\left(\frac{{\text{D}}_{\text{a}}\text{t}}{{\text{L}}^{2}}\right)}+\frac{1}{25}{\text{e}}^{-25\left(\frac{{\text{π}}^{2}}{4}\right)\left(\frac{{\text{D}}_{\text{a}}\text{t}}{{\text{L}}^{2}}\right)}\right)+\cdots$$

Discarding the higher order terms in the equation, assuming C_∞_ = 0, and regrouping the terms, this equation was simplified into Eq. (3):

(3) $$-\text{ln}\left(\frac{\pi^{2}{\text{C}}_{\text{t}}}{8{\text{C}}_{\text{i}}}\right)=\frac{{\text{π}}^{2}{\text{D}}_{\text{a}}\text{t}}{{4\text{L}}^{2}}$$

where Ct = moisture content at time t (kg H_2_O/kg solids); Ci = initial moisture content (kg H_2_O/kg solids); t = time (min); L = thickness (m); Da = diffusion coefficient m^2^/s^−1^.

Activation energy can be interpreted as the energy required to produce the diffusive motion of one mole of atoms. The influence of the frying temperature on the effective diffusion coefficient had an Arrhenius-type behavior (Alvis et al., 2010), as shown with Eq. (4):

(4) $${\text{D}}_{\text{a}}{=\text{D}}_{\text{0}}{\text{exp}}^{\left(\frac{{-\text{E}}_{\text{a}}}{\text{RT}}\right)}$$

where, E_a_ = Activation energy; T = absolute temperature; Di = Reference diffusion coefficient. R = Universal gas constant (8.314 × 10^−3^ kJ/mol ° K).

Eq. (5) was used to determine the activation energy (Ea in kJ/mol), linearize, graph ln Da vs 1/T and estimate E_a_ from the value of the slope:

(5) $${\text{LnD}}_{\text{a}}={\text{lnD}}_{\text{i}}\frac{{-\text{E}}_{\text{a}}}{\text{R}}\frac{1}{\text{T}}$$

The porosity of the fried yam was calculated using the ratio of the real density and the apparent density with Eqs. (6) and (7). A 1000 ml test tube was used, which was filled with water up to 500 ml. Then, a yam that was previously weighed was introduced, thereby determining the real volume of the yam by displacing the volume of water and using Eq. (6). Then, a cardboard box measuring 0.34 × 0.2 × 0.2 m was used. In each observation, yams of the same grade and variety were randomly introduced, and their weight was determined (equation 7) (Buitrago et al., 2004). With these data, the porosity was determined with Eq. (8):

(6) $$\text{Real ​Density}\;\left({\text{γ}}_{\text{r}}\right)\;\left(\;\frac{\text{ ​ ​Product ​weight ​}}{\text{ ​ ​Real ​product ​volume}}\right)$$

(7) $$\text{Apparent ​Density}\left({\text{γ}}_{\text{a}}\right)=\left(\frac{\text{Weight ​}}{\text{Volume ​}}\right)$$

(8) $$\text{Porosity}=\left(1-\frac{{\text{γ}}_{\text{Apparent}}}{{\text{γ}}_{\text{Real}}}\right)\text{x}100$$

where, Weight: weight of the product in the container; Volume: volume of the container.

## 3 Results and discussion

The results obtained from the characterization of the four varieties of yams grown on the Colombian Atlantic Coast are shown in Table 1, which shows that this food has a higher proportion of water and carbohydrates and is a moderate source of energy (Blanco-Metzler et al., 2004). These results are very similar to those obtained by (Alvis et al., 2008). In these results, only trace levels of fat were detected, much lower than those reported by (Wanasundera and Ravindran, 1992) but very similar to those of (Alvis et al., 2008). On the other hand, the total carbohydrate content was lower than those reported by (De Paula et al., 2012). The Latin American food composition table (LATINFOODS, 2010) contains values similar to those obtained in this research, except for protein, fat and ash, which for these varieties were lower.

**Table 1:** Proximate composition of Yam.

Factor	Variety of yams			
	125 Espino Brasilero	153 Espino Traditional	619 Espino African	091 Espino
Moisture	67.39 ± 0.41^a^	70.61 ± 0.36^b^	71.67 ± 0.24^c^	66.73 ± 0.45^a^
Crude fat	0.09 ± 0.01^d^	0.05 ± 0.01^b^	0.03 ± 0.01^a^	0.05 ± 0.01^b^
Crude protein	1.09 ± 0.12^d^	0.78 ± 0.02^a^	0.96 ± 0.00^b^	0.95 ± 0.01^b^
Ash	0.37 ± 0.01^a^	0.34 ± 0.04^a^	0.23 ± 0.01^b^	0.21 ± 0.03^b^
Crude fiber	0.14 ± 0.02^a^	0.19 ± 0.04^c^	0.17 ± 0.04^b^	0.17 ± 0.04^b^
Total sugar	31.06 ± 0.47^b^	28.21 ± 0.33^a^	27.11 ± 0.22^a^	32.06 ± 0.46^b^
Reducing sugar	P90.20 ± 0.00^a^	0.23 ± 0.00^b^	0.59 ± 0.01^d^	0.41 ± 0.00^c^

∗ Mean values identified by the same letter in the row do not differ significantly from each other.

Of the four varieties analyzed, higher reducing sugar contents were observed in 619 African Espino, with 0.59 ± 0.01%, and 091 Espino, with 0.41 ± 0.00%. The former had the highest sugar content. A low sugar content was observed in 125 Brazilian Espino, with 0.20 ± 0.00%, and 153 Traditional Espino, with 0.23 ± 0.00%. These results are shown Table 1, confirming that sugars cause browning during frying as a result of their reaction with the amino group of proteins (Alvis et al., 2008).

### 3.1 Moisture loss and oil absorption

**Figure 1:**
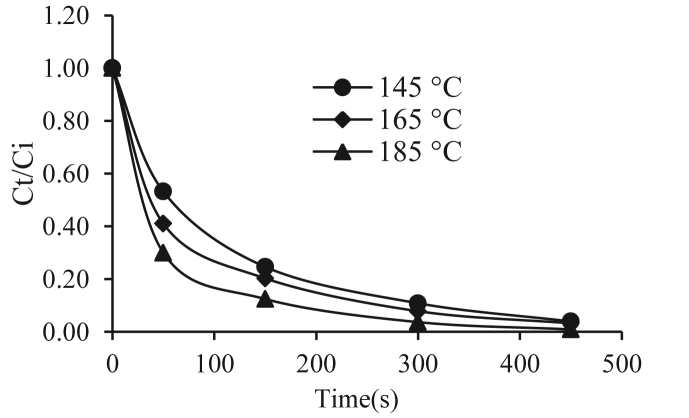
Dimensionless humidity Vs time Traditional Espino.

**Figure 2:**
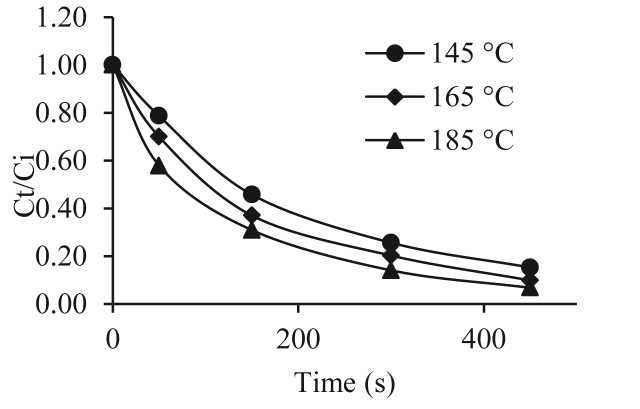
Dimensionless moisture Vs time for Brazilian Espino.

Figures 1 and 2 show the moisture values (as dimensionless humidity *Ct/Ci*), where *Ct* is the moisture content on a dry basis at time t, and *Ci* is the Initial moisture content on dry basis during the immersion frying process at 145, 165 and 185 °C for the varieties 153 Traditional Espino and 125 Brazilian Espino. In the two varieties, the frying process included an initial stage in which the water loss was high, mainly the loss of surface water. During this stage, the water vapor is released uniformly across the entire surface as small bubbles. Then, at the beginning of the crust formation stage, the evaporation rate decreases until evaporation is relatively constant for the final frying times (Moya, 2011; Alvis et al., 2015).

When comparing the varieties, for up to 2 min of frying (initial stage), the rate of water loss in the 153 Traditional Espino variety was greater than in the 125 Brazilian Espino variety.

During the initial frying period, a greater loss of moisture was observed, which tended to stabilize after 5 min because of evaporation from the surface of the product. The effect of temperature on water loss has already been studied and reported in previous research (Moyano et al., 2005; Pedreschi et al., 2007). These authors found that the rate of moisture loss is higher at high frying temperatures because the evaporation rate is increased. It has been observed that there is greater evaporation of water at 185 °C than at 145 and 165 °C for both varieties.

**Figure 3:**
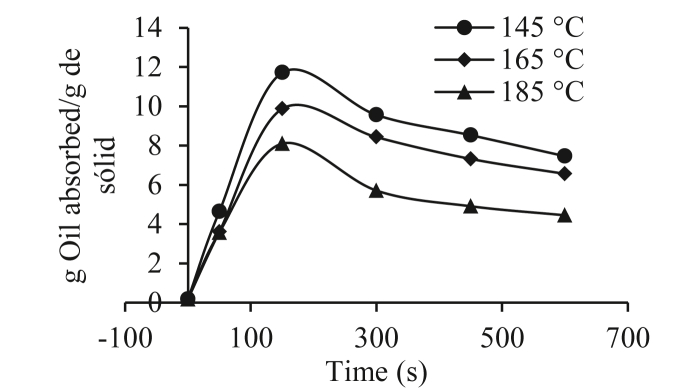
Oil absorption during frying of the 153 Traditional Espino variety.

**Figure 4:**
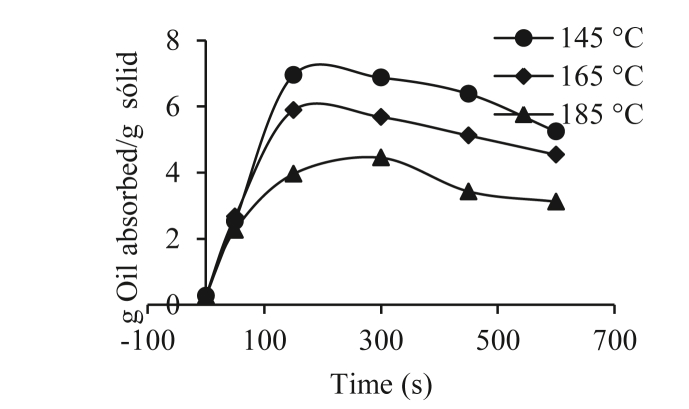
Oil absorption during frying of variety 125 Brazilian Espino.

Figures 3 and 4 show the oil content (g oil absorbed/g solids) over time (seconds) for the yam varieties at the different frying temperatures of 145, 165 and 185 °C. Figure 3 shows that the absorption of oil was very high at the beginning of the frying, for which numerous studies have suggested that the absorption of oil is a surface phenomenon that depends mainly on the microstructure of the crust that is forming (Mellema, 2003; Moyano and Pedreschi, 2006; Troncoso and Pedreschi, 2009). It should be noted that, during the frying process, most of the oil remains on the surface of the yam and is mainly absorbed when the pieces of yam are removed from the frying medium, that is, in the post-frying, cooling period (Bouchon et al., 2003; Troncoso and Pedreschi, 2009). During the frying process, it can be observed that the oil content increased, then tended to decrease until it reached equilibrium, which represented the maximum amount of oil that the piece of fried yam could absorb.

Figure 4 shows the same oil absorption behavior for the 153 Traditional Espino variety, but less oil was absorbed. In general terms, this result agrees with the fact that a higher frying temperature leads to lower oil absorption (Rioseco, 1999; Pedreschi and Moyano, 2005; Alvis et al., 2010). For both varieties, it was observed that the oil content increased with the process time, reaching a maximum at 190 s in the 153 traditional hawthorn and at 200 s for the 125 Brazilian hawthorn at a temperature of 145 °C for both varieties, which was attributed to the replacement of evaporated water. Some authors have reported an increase in the oil content of corn tortillas (Moreira et al., 1995), potatoes (Math et al., 2004), pieces of potatoes (Yildiz et al., 2007), pieces of yam (Alvis et al., 2010), and pieces of sweet potato (Alvis et al., 2015).

### 3.2 Mass diffusion coefficient during immersion frying

(Da) was estimated from the value of the slope with Eq. (3) for the three frying temperatures: 145, 165 and 185 °C for the two yam varieties (Table 2). First, it was observed that there were clear differences between the studied yam varieties. The 125 Brazilian Espino variety presented higher values of effective diffusivity than the 153 Traditional Espino variety.

**Table 2:** Effective diffusion coefficient and activation energy in yam frying.

T (°C)	153 Traditional Espino		125 Espino Brasilero	
	D_a_ (m^2^. s^−1^ ∗10^−8^)	Ea (kJ/mol)	D_a_ (m^2^s^−1^ ∗10^−8^)	Ea (kJ/mol)
145	6.99 ± 0.78	13.61	4.26 ± 0.63	12.86
165	8.71 ± 0.82		5.07 ± 0.67	
185	9.83 ± 0.84		5.67 ± 0.68	

The effective diffusivity for both varieties represented the rate at which the water left the yam pieces and diffused into the oil during the frying process. It was assumed that, at a higher temperature, the absorption of oil in a product decreases (Moyano and Pedreschi, 2006); therefore, the diffusion coefficient decreases as a crust forms on the surface of the product, which makes diffusion slower. The results indicated that a higher frying temperature (185 °C) results in a higher effective diffusion coefficient. This behavior occurred in both yam varieties. The Da values varied between 4.26 × 10^−8^ m^2^. S^−1^ and 5.67 × 10^−8^ m^2^ s^−1^ for the 125 Espino Brazilian variety and between 6.99 × 10^−8^ m^2^ s^−1^ and 9.83 × 10^−8^ m s^−1^ for the 153 Espino Traditional variety (Table 2), values that are in the normal expected range of 10–12 to 10^−8^ m^2^ s^−1^ for dehydrated foods (Giovanelli et al., 2002; Gastón et al., 2004; Gelly and Santalla, 2007) and foods obtained with other methods (Math et al., 2004; Akanbi et al., 2006; Nguyen et al., 2006). The (Da) value during the drying of a given food depends on the procedure types and experiment conditions used to determine the water content, the methods used to treat the data, and the heterogeneity of the structures (Corzo et al., 2011).

The activation energy value (Table 2) for variety 153 Espino Traditional was 13.61 kJ/mol and, for variety 125 Espino Brazilian, was 12.86 kJ/mol, which was within the range of values found in the literature for food materials (12.7–110 kJ/mol): 45 kJ/mol for potato (Math et al., 2004); 28.39 kJ/mol for carrot (Doymaz, 2004); 21.49 kJ/mol for sliced yam *D. rotundata* 9811-089 (Montes et al., 2008); 17.50 kJ/mol for *D. rotundata* 9811-091 yam fillets (Montes et al., 2008); 24.43 kJ/mol for Diamond 22 yam (Alvis et al., 2010), and 21.27 kJ/mol for (Pico Botella) yams (Alvis et al., 2010). These activation energy values represented the energy required to initiate the diffusion of moisture from *D. rotundata* during moisture loss and indicated the influence of temperature in the process (Montes et al., 2008). High activation energy (Ea) values indicate the high sensitivity of the effective diffusion coefficient to temperature. A process controlled by diffusion has an activation energy lower than 34 kJ/mol (Corzo et al., 2011); therefore, the estimated (Ea) values suggested that the diffusion of water in the pieces of yam was the limiting mechanism in the moisture loss.

### 3.3 Determination of porosity

When the frying process begins, the surface temperature of the food rises rapidly, and the surface water begins to boil. Because of the evaporation of water, the food surface dries. In addition to leading to contraction and the development of surface porosity and roughness, there is a smaller production of bubbles as a consequence of the decrease in surface moisture, progressively generating the formation of a crust (Rioseco, 1999; Mellema, 2003). Then, with the formation of a crust as the frying process progresses, porosity decreases and prevents the rapid exit of water (Berna, 1999). Table 3 shows the results for the porosity of the fried yams (parallelepipeds, 1 × 1 × 4 cm) at temperatures of 145, 165 and 185 °C with the final frying time, according to the methodology of Buitrago et al. (2004).

**Table 3:** Porosity of fried yams.

T (°C)	Porosity	
	153 Espino Traditional	125 Espino Brasilero
145	62.26 ± 0.58	50.98 ± 0.57
165	64.17 ± 0.48	59.14 ± 0.53
185	66.21 ± 0.52	60.31 ± 0.60

As can be seen, if moisture loss is high, the porosity increases during frying until reaching a balance in the final moments of the process. The greater the water loss from the food, the greater the porosity is. This parameter is important in cell diffusion during frying, so this behavior may suggest that frying at high temperatures results in greater facility for water to leave the product tissue as a result of an increase in porosity (Moya, 2011).

## 4 Conclusion

The selected yam varieties were Espino Brazilian and 153 traditional espino because they presented a low content of reducing sugars during the characterization. There was greater evaporation of water at 185 °C than at 145 or 165 °C for both varieties. The traditional hawthorn variety lost the most water. At higher temperatures there was less oil absorption, where the Brazilian hawthorn variety absorbed the least amount of oil. The moisture loss and oil gain in the parallelepiped-shaped pieces of yam was described with Fick's second law for infinite plate geometry. As the temperature increased, the effective diffusivity increased according to the Arrhenius equation, where the 125 Espino Brazilian variety presented higher effective diffusivity values than the 153 Traditional Espino variety. The porosity of the fried yam pieces was higher as the temperature increased in both yam varieties, but the values were higher for the 153 traditional Espino variety.

## Declarations

### Author contribution statement

Arrazola Guillermo: Conceived and designed the experiments; Performed the experiments; Contributed reagents, materials, analysis tools or data; Wrote the paper.

Alvis Armando: Performed the experiments; Analyzed and interpreted the data; Wrote the paper.

Romero Pedro: Analyzed and interpreted the data; Wrote the paper.

### Funding statement

This research did not receive any specific grant from funding agencies in the public, commercial, or not-for-profit sectors.

### Data availability statement

Data included in article/supplementary material/referenced in article.

### Declaration of interests statement

The authors declare no conflict of interest.

### Additional information

No additional information is available for this paper.
